# *Legionella feeleii*: Ubiquitous Pathogen in the Environment and Causative Agent of Pneumonia

**DOI:** 10.3389/fmicb.2021.707187

**Published:** 2021-08-03

**Authors:** Lucianna Vaccaro, Thiago Santos Gomes, Fernando Izquierdo, Angela Magnet, Sergio Llorens Berzosa, Dolores Ollero, Santiago Salso, Almudena Alhambra, Carmen Gómez, María López Cano, Carmen Pelaz, Beatriz Bellido Samaniego, Carmen del Aguila, Soledad Fenoy, Carolina Hurtado-Marcos

**Affiliations:** ^1^Departamento Ciencias Farmacéuticas y de la Salud, Facultad de Farmacia, Universidad San Pablo-CEU, CEU Universities, Madrid, Spain; ^2^Coordenação de Aperfeiçoamento de Pessoal de Nivel Superior (CAPES) Foundation, Ministry of Education of Brazil, Brasília, Brazil; ^3^Hospital Universitario HM Monteprincipe y Sanchinarro, Madrid, Spain; ^4^Unidad de Legionella, Laboratorio de Referencia e Investigación en Infecciones Bacterianas Transmitidas por Agua y Alimentos, Centro Nacional de Microbiología (CNM), Instituto de Salud Carlos III (ISCIII), Madrid, Spain

**Keywords:** *Legionella*, *L. feeleii*, Legionnaires’ disease, molecular diagnosis, drinking water treatment plants

## Abstract

*L. feeleii* is one of the most frequent *Legionella* species isolated from natural pools of the central region of Spain. This study aimed to evaluate its ecology and to identify this *Legionella* species as a respiratory pathogen. A PCR assay for detecting the *L. feeleii* mip gene was developed to identify it in clinical and environmental samples. Culture and PCR were performed in environmental samples from four drinking water treatment plants (DWTPs). Free *L. feeleii* was only detected in raw water samples (3.4%), while *L. feeleii* as an *Acanthamoeba* endosymbiont was found in 30.7% of raw water, 11.5% of decanter biofilm, and 32% of finished water samples. Therefore, *Acanthamoeba* spp. plays an essential role in the multiplication, persistence, and spread of *Legionella* species in the environment. The first case of Legionnaires’ disease caused by *L. feeleii* in Spain is described in this study. The case was diagnosed in an older woman through PCR and sequencing from urine and sputum samples. A respiratory infection could be linked with health care procedures, and the patient presented several risk factors (age, insulin-dependent diabetes, and heart disease). The detection of non-*L*. *pneumophila*, such as *L. feeleii*, is a factor that must be considered when establishing or reviewing measures for the control and prevention of legionellosis.

## Introduction

*Legionella*, a causative agent of pulmonary infection, is ubiquitous in aquatic ecosystems. It was first described from an outbreak of pneumonia in Philadelphia (United States) during the American Legion convention in 1976 (Fraser et al., 1977).

This Gram-negative bacillus is one of the most commonly associated with severe pneumonia and is responsible for significant morbidity and mortality, particularly in immunosuppressed and elderly individuals. The case-fatality rate can reach up to 20% in community-acquired pneumonia and more than 40% in nosocomial infection (Bartram et al., 2007; Ishiguro et al., 2013; Phin et al., 2014).

Legionellosis is a notifiable disease that has been reported worldwide (Bartram et al., 2007). About 10–15 cases were detected per million people in Europe, Australia, and United States (World Health Organization (WHO), 2016). In Europe, Spain has reported one of the highest numbers of legionellosis cases in recent years, being ranked third in 2015 with more than 1,000 confirmed cases (European Centre for Disease Prevention and Control, 2017).

The main route of transmission is by inhalation of aerosols or aspiration of water contaminated with *Legionella*. The most common sources of infection are cooling towers, evaporative condensers, decorative fountains, thermal baths, hospital facilities, and respiratory therapy equipment (Eickhoff, 1979; Mercante and Winchell, 2015; Cunha et al., 2016; Kanamori et al., 2016). Infection occurs in susceptible individuals, who may develop a mild and self-limiting illness (non-pneumonic) known as Pontiac Fever, or a severe and sometimes fatal form of pneumonia called Legionnaires’ disease (Bartram et al., 2007).

The survival and multiplication of *Legionella* in the environment are mainly due to its ability to associate with complex biofilm communities and to replicate inside free-living amoebae (FLA) as an intracellular pathogen, although it can also be free-form in aquatic ecosystems (Winiecka-Krusnell and Linder, 1999; Taylor et al., 2009). These bacteria are often found in low concentrations in nature and water purification facilities such as drinking water treatment plants (DWTPs). However, these bacteria can reach potable water distribution systems and colonize water plumbing systems (Mercante and Winchell, 2015). Therefore, the presence of *Legionella* in nature and DWTPs needs to be evaluated to understand the relevance of these ecological niches for the prevalence of these bacteria in drinking water distribution systems.

Currently, there are over 60 species, three (3) subspecies, and more than 78 serotypes of *Legionella* described in the literature (Euzeby, 1997). *Legionella feeleii* is one of the most frequent *Legionella* species isolated from natural pools of the central region of Spain (Magnet et al., 2015). Although *L. feeleii* has yet to be reported as a causative agent of pneumonia in this country, it could represent a risk factor to a population exposed to these sources (Lee et al., 2009; Cervero-Aragó et al., 2015). Taking into account its environmental presence, the lack of any reported case could be due to the fact that the diagnostic methods most commonly used in hospitals to confirm cases of Legionnaires’ disease (immunochromatography and ELISA) only detect the *L. pneumophila* serogroup 1 (Muder and Yu, 2002; Blyth et al., 2009). Thus, infections caused by *L. feeleii* or other species of non-*L*egionella *pneumophila* are not detected (Muder and Yu, 2002; Svarrer et al., 2012; Vaccaro et al., 2016; Chambers et al., 2021).

The gold standard technique (isolation by culture) and pioneering methods (immunofluorescence) for the diagnosis of legionellosis are rarely used nowadays (Cunha et al., 2016; European Centre for Disease Prevention and Control, 2017). In contrast, molecular tools are increasingly being developed to identify cases of legionellosis due to their higher sensitivity and specificity (Cloud et al., 2000). A comparative study for *Legionella* detection in urine samples demonstrated that a large number of cases were identified by PCR assay rather than by antigen immunochromatography test (Vaccaro et al., 2016). Therefore, there is a clear need for molecular diagnostic techniques in hospitals to identify *Legionella* species associated with this disease. In Denmark and New Zealand, PCR protocols are already being employed as routine diagnostic methods (Svarrer and Uldum, 2012; Murdoch et al., 2013; European Centre for Disease Prevention and Control, 2017; Chambers et al., 2021). Some of the most common targets for molecular diagnosis include 16S rRNA and *mip* (macrophage infectivity potentiator) genes of *Legionella* spp. (Miyamoto et al., 1997; Ratcliff et al., 1997; Haroon et al., 2012; Svarrer and Uldum, 2012).

Therefore, the aims of this study were, (i) to develop a PCR assay in order to detect the *mip* gene of *L. feeleii* as an alternative molecular method for environmental and clinical samples and (ii) to use it to verify the possible presence of this *Legionella* species as a respiratory pathogen, as well as an environmental contaminant of waters from DWTPs in the central area of Spain.

## Materials and Methods

### Bacteria, Culture, and DNA Extraction

Nine *Legionella* strains (*L. feeleii, L. anisa, L. bonzemanii, L. dumoffi, L. jordanis, L. longbeachae, L. micdadei, L. pneumophila* segrogroup 1, *L. pneumophila* serogroup 2–14) were used in this study. All strains were cultured on *Legionella* selective medium GVPC at 37°C for 3–12 days. Other respiratory pathogens were cultured on their specific agars such as blood-agar media, MacConkey, cetrimide, chocolate and sabouraud agar. From Gram-negative bacteria, genomic DNA was extracted by heat shock (99°C for 20 min) and purified using the NucleoSpin^®^ Gel and PCR Clean-up extraction kit (Macherey-Nagel, Germany). Gram-positive bacteria and fungal pathogens genomic DNA were extracted using the NucleoSpin^®^ Genomic DNA from tissue kit (Macherey-Nagel, Germany), according to the manufacturer’s instructions. The genomic DNA was eluted with 100 μL of Milli-Q^®^ water and stored at −80°C until PCR assay.

### PCR Protocol

(i) **Oligonucleotide primers:** Lmip_F (5′-GGCAATGTC AACGACAATTGC-3′) and Lmip_R (5′-TGGCCAT CAATTAACTTGCCAGT-3′) were designed from partial amplification of the *mip* gene of *L. feeleii* serogroups 1 and 2. Primers amplified from 417 to 863-bp from Genebank sequence accession numbers U92205 and AF022341.
  An *in silico* analysis through Bioedit 7.2 was performed to evaluate the binding of the primers Lmip_F and Lmip_R with the DNA sequences of the *mip* gene *Legionella* species (Supplementary Figure 1). These sequences were taken from the GenBank database.
(ii) **PCR conditions:** Five microliter of extracted DNA was used in 20 μL reaction mixture that included 0.5 μL of each primer 20 μM, 9 μL of Milli-Q^®^ water, and 10 μL of 2X Phusion flash high-fidelity PCR master Mix (Thermo Scientific^TM^, United States). Thermal cycling was performed with a GeneAmp^®^ PCR System 9700 (Applied Biosystems, United States), and cycling conditions were: 1 cycle at 98°C for 15 s, 35 cycles at 98°C for 1 s, 60°C for 5 s and 72°C for 15 s, with a final extension at 72°C for 1 min.
(iii) **Detection of amplified products:** PCR products were visualized by electrophoresis in 2% agarose gels with ethidium bromide staining (0.5 μg/mL). DNA fragments of 447-bp were purified using NucleoSpin^®^ Gel and PCR Clean-up extraction kit (Macherey-Nagel, Germany), following the manufacturer’s instructions.
(iv) **Sequencing:** Purified PCR amplicons were sequenced at both ends with PCR primers through Macrogen laboratories (South Korea) sequencing service. The sequences were analyzed with Bioedit Sequence Alignment Editor 7.2 and were confirmed by similarity using the BLAST (Basic Local Alignment Search Tool).
(v) **Control material:** An environmental strain of *L. feeleii* (collection of environmental bacteria from San Pablo CEU University, Magnet et al., 2015) was used as a positive control. Milli-Q^®^ water was used as a negative control. Milli-Q^®^ water used for DNA extraction, the PCR reaction mixture, and the negative control were previously treated with ultraviolet light.

### Collection of Environmental Samples From DWTPs

**FIGURE 1:**
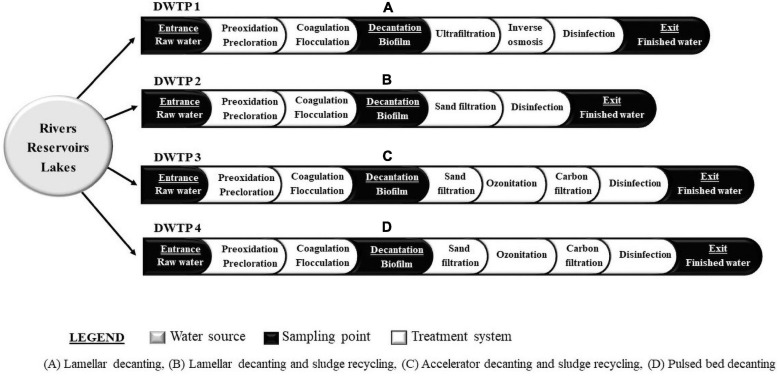
Schematic representation of water treatment lines of drinking water treatment plants (DWTPs) sampled. **(A)** Lamellar decanting. **(B)** Lamellar decanting and sludge recycling. **(C)** Accelator decanting and sludge recycling. **(D)** Pulsed bed decanting.

A total of 87 samples of raw water, finished water, and decanter biofilm was collected from four DWTPs located in the central region of Spain (Figure 1). All points studied were sampled twice each season during 2014–2015.

#### Collection of Water Samples

Samples were obtained using two methods: (i) 1 L of water was collected in a sterile plastic bottle and concentrated following ISO standard 11,731-2:2004 method (Water Quality-Detection and enumeration of *Legionella*); (ii) 50 L of water were collected and concentrated using IDEXX^®^ Filta Max system, according to the manufacturer’s instructions.

#### Collection of Decanter Biofilm

Samples were collected using a sterile swab. Subsequently, these swabs were suspended in 500 μL of PBS 1X (Phosphate Buffered Solution).

### Detection of *L. feeleii* in Environmental Samples From DWTPs

**FIGURE 2:**
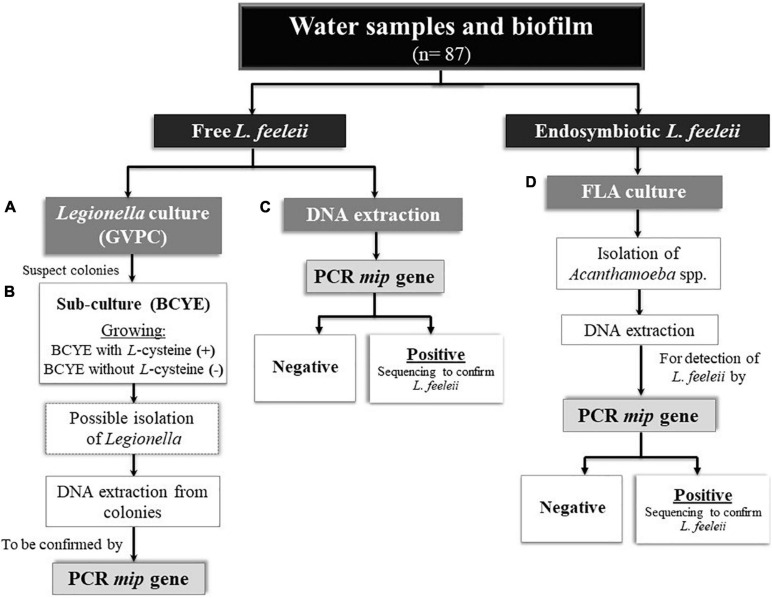
Microbiological and molecular detection of *L. feeleii* in environmental samples from drinking water treatment plants (DWTPs). **(A)** Selective medium GVPC for the primary isolation of *Legionella*. **(B)** BCYE medium with and without *L*-cysteine to confirm *Legionella* isolated. **(C)** Genomic DNA extraction from the concentrated water sample and homogenized biofilms. **(D)** 2% Neff’s saline non-nutrient agar plates seeded with heat shock inactivated *Escherichia coli.* FLA, free-living amoebae.

All concentrated samples were analyzed to detect *L. feeleii* by culture isolation and PCR (Figure 2).

#### Free *Legionella feeleii*

##### Legionella Culture

Hundred microliter of each environmental sample were cultured in selective medium GVPC plates and incubated at 37°C for 10–12 days. These cultures were monitored periodically. Suspect colonies were incubated in buffered charcoal yeast extract agar (BCYE) with and without *L-*cysteine and confirmed as *Legionella* spp. If they necessitated *L*-cysteine to grow. *Legionella* colonies were then suspended in 100 μL of PBS 1X, and DNA was extracted by heat shock (99°C for 20 min). The genomic DNA was purified using NucleoSpin^®^ Gel and PCR Clean-up extraction kit (Macherey-Nagel, Germany) and eluted in 50 μL Milli-Q^®^ water. The extracted DNA was stored at −80°C until analysis.

##### DNA Extraction From Water Samples

Genomic DNA extraction was performed with FastDNA^TM^ kit (Biomedicals, France) from 200 μL of each concentrated water sample and homogenized biofilms, modifying the manufacturer’s protocol. Briefly, to each Fastprep tube another 1/4-inch ceramic sphere was added, and lysing cycles were performed in triplicate. The extracted DNA was stored at −80°C until PCR assay.

#### Endosymbiont *Legionella feeleii* Associated With FLA

##### FLA Culture

Eighty microliter of environmental samples were inoculated onto 2% Neff’s saline non-nutrient agar plates seeded with heat shock inactivated *Escherichia coli*. The plates were incubated at 28°C for 14–20 days. Isolation of *Acanthamoeba* spp. was performed according to the protocol described by Magnet et al. (2012).

##### DNA Extraction From Acanthamoeba Isolates

Genomic DNA was extracted from 100 μL of diluted FLA culture by heat shock (99°C for 20 min) and purified using NucleoSpin^®^ Gel and PCR Clean-up extraction kit (Macherey-Nagel, Germany). DNA elution was performed with 50 μL Milli-Q^®^ water and stored at −80°C until PCR assay.

### Clinical Report

#### Clinical Diagnosis

A case of atypical pneumonia was identified according to criteria established by the Spanish Society of Pneumology and Thoracic Surgery (SEPAR) (Menéndez et al., 2010; Blanquer et al., 2011).

#### Laboratory Diagnosis

Urine and sputum samples were collected from a patient with pneumonia.

(i) ***Legionella* and *Streptococcus pneumoniae* by urinary antigen test:** The urine sample was analyzed with *Legionella* urinary antigen card kit and *Streptococcus pneumoniae* antigen card kit (Alere BinaxNow^®^, United States) following the manufacturer’s instructions. These commercial kits, immunochromatography tests, are commonly used in hospitals for the qualitative detection of *L. pneumophila* serogroup 1 and pneumococcal infection.
(ii) **Sputum culture:** An optimal specimen was graded using the Murray and Washington system (Winn et al., 2007). The sputum sample was inoculated on: (i) *Legionella* selective medium GVPC plate/incubation at 37°C for 10–12 days, (ii) blood agar and chocolate agar/incubation at 37°C in CO_2_ (5–7%) for 2 days.
(iii) **DNA extraction from sputum and urine samples:** Genomic DNA was extracted from both samples by NucleoSpin^®^ Genomic DNA from tissue kit (Macherey-Nagel, Germany), following the manufacturer’s instructions. DNA was eluted in 50 μL Milli-Q^®^ water and stored at −80°C until PCR assay.
(iv) **Identification of *L. feeleii* DNA and sequencing confirmation:** The PCR assay developed in this study was performed for specific detection of *L. feeleii* DNA. Additionally, a semi-nested PCR described by Miyamoto et al. (1997) was used to amplify the 16S rRNA gene of *Legionella* spp. partially. Positive controls were genomic DNA of *L. pneumophila* serogroup 1 (NCTC12821) and *L. feeleii* [collection of environmental bacteria from San Pablo CEU University (Magnet et al., 2015)].

### Ethics Statement

#### Environmental Samples

A collaboration agreement was signed for research purposes between the competent authority of DWTPs and the San Pablo CEU University (Laboratory of Parasitology, Faculty of Pharmacy). We were authorized to sample raw water, finished water, and decanter samples to detect *Legionella* and FLA.

#### Clinical Samples

This clinical research was carried out in compliance with fundamental ethical principles, including those set out in the Charter of Fundamental Rights of the European Union and the European Convention on Human Rights. The patient attested her agreement to participate in this epidemiological survey through a written informed consent, which was evaluated and approved by the Research Ethics Committee of San Pablo CEU University, following the recommendations of the Spanish Bioethics Committee, the Spanish legislation on Biomedical Research (Law 14/2007, of July 3rd) and Personal Data Protection (Organic Law 15/1999 and Royal Decree 1720/2007). The patient’s identification data was kept on a condition of anonymity.

## Results

### Conventional PCR Assays Targeting the *mip* Gene

In the present study, a conventional PCR assay was developed to detect the *mip* gene of *L. feeleii.* A specific 447-bp DNA fragment amplified by Lmip primers was observed in 0.05 pg of *L. feeleii* DNA using the present PCR protocol (Supplementary Figure 2A). This pair of primers also allowed the simultaneous detection of *L. micdadei* with a PCR product size of 900-bp (Supplementary Figure 2B and Table 1).

**TABLE 1:** Results of the optimization of *Legionella* species detection with Lmip primers.

**Species *Legionella***	**Strain**	**PCR product (bp)**	**Limit of detection** **of DNA (ng)^a^**
*L. feeleii*	From an environmental specimen^b^	447*	5 × 10^–5^
*L. micdadei*	ATCC 33218	900	5 × 10^–3^
*L. anisa*	ATCC 35292	Undetected	NA
*L. bonzemanii*	ATCC 33217	Undetected	NA
*L. dumoffii*	ATCC 33279	Non-specific** (1000-bp)	NA
*L. jordanis*	ATCC 33623	Undetected	NA
*L. longbeachae*	ATCC 33462	Non-specific** (700-bp)	NA
*L. pneumophila* sg. 1	NCTC 12821	Undetected	NA
*L. pneumophila* sg. 2–14	From an environmental specimen^c^	Undetected	NA

a) Minimum concentration of detected Legionella DNA. b) This environmental strain of L. feeleii was isolated by Magnet et al. (2015). c) This environmental strain of L. pneumophila sg. 2–14 was isolated by Salinas et al. (2021). *In silico analysis revealed a similar size for *Legionella tunisiensis* amplicon. Sequencing analysis should be carried out to distinguish both species. **L. dumoffii and L. longbeachae, when loaded with a high concentration (50–100 ng), might show a weak amplification of a non-specific target. Sg., serogroup; NA, non-applicable; ATCC, American Type Culture Collection; NCTC, National Collection of Type Cultures.

The *in silico* analysis with other *Legionella* species revealed that the expected amplicon for *Legionella tunisiensis* with the described primers would be similar in size (447-bp) to that of *L. feeleii* (Supplementary Figure 3). Therefore, amplicons of that size should be sequenced in order to determine the species.

The evaluation of the specificity of this PCR protocol was also performed for other respiratory pathogens and environmental microorganisms (Supplementary Table 1). This evaluation indicated *Streptococcus pneumoniae* amplifies a 450-bp fragment, with a similar length of the fragment amplified with *L. feeleii* DNA. However, the PCR products presented a double-band pattern of amplification with those bacteria, with fragments of approximately 450 and 800-bp, while *L. feeleii* only presented the amplification of a single fragment of 447-bp. On the other hand, *L. longbeachae* and *L. dumoffii*, when loaded with a high concentration (50–100 ng), might show a weak amplification of a non-specific target of 700 and 1,000-bp, respectively (Supplementary Figure 2C).

### Presence of *L. feeleii* in DWTPs

**FIGURE 3:**
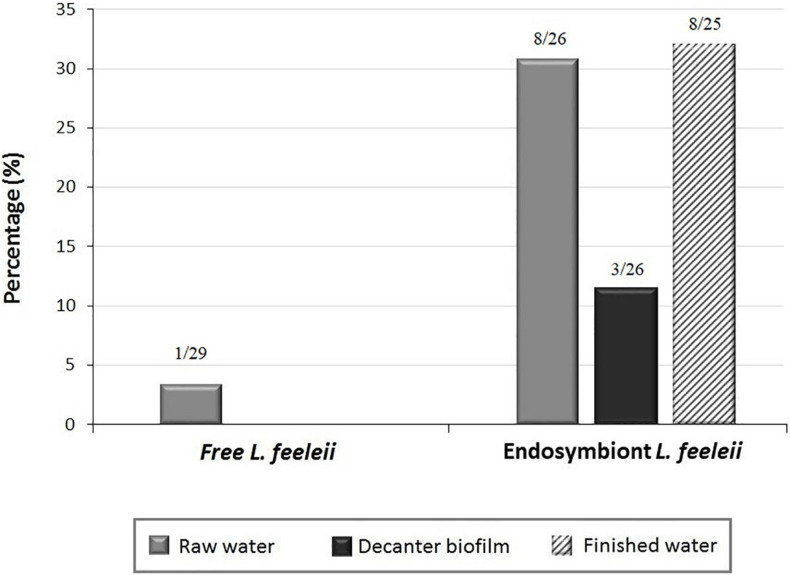
Presence of *L. feeleii* in environmental samples from drinking water treatment plants (DWTPs).

The detection of *L. feeleii* was carried out using the PCR assay targeting the *mip* gene developed in the present study. Figure 3 shows the frequency of *L. feeleii* detected in environmental samples from 4 DWTPs of the central region of Spain. Free *L. feeleii* was only detected in raw water samples (1 out of 29 samples, 3.4%). On the other hand, *L. feeleii* as an *Acanthamoeba* endosymbiont was found in 30.7% of raw water (8 out of 26 samples), 11.5% of decanter biofilm (3 out of 26 samples), and 32% of finished water samples (8 out of 25 samples). Table 2 contained the results classified according to the detection method, DTWPs, seasons, sampling point, and *Legionella* state (free-living or endosymbiont).

**TABLE 2:** Presence of *L. feeleii* in each sampling point from 4 DWTPs of the central region of Spain according to detection methods.

**Samples and analysis**	**DWTP1**					**DWTP2**					**DWTP3**					**DWTP4**				
	**W**	**Sp**	**S**	**A**	**Total (%)**	**W**	**Sp**	**S**	**A**	**Total (%)**	**W**	**Sp**	**S**	**A**	**Total (%)**	**W**	**Sp**	**S**	**A**	**Total (%)**
**Raw water**																				
Agar Culture	−/−	−/−	+/NC	NC	20	−/−	−/−	−/−	−/−	0	−/−	−/−	−/−	−/−	0	−/−	−/−	−/−	−/−	0
Direct PCR on water	−/−	−/−	+/NC	NC	20	−/−	−/−	−/−	−/−	0	−/−	−/−	−/−	−/−	0	−/−	−/−	−/−	−/−	0
PCR in isolated amoebae	−/−	−/−	+/NC	NC	20	−/−	−/−	+/+	±	37.5	−/+	−/+	±	±	50	−/−	NA/NA	−/−	NA/+	20
**Decanter biofilm**																				
Agar Culture	−/−	−/−	−/NC	NC	0	−/−	−/−	−/−	−/−	0	−/−	−/−	−/−	−/−	0	−/−	−/−	−/−	−/−	0
Direct PCR on water	−/−	−/−	−/NC	NC	0	−/−	−/−	−/−	−/−	0	−/−	−/−	−/−	−/−	0	−/−	−/−	−/−	−/−	0
PCR in isolated amoebae	NA/−	−/−	−/NC	NC	0	−/−	−/+	−/−	−/−	12.5	−/−	±	−/NA	NA/+	33.3	−/−	−/−	−/−	−/−	0
**Finished water**																				
Agar Culture	−/−	−/−	−/NC	NC	0	−/−	−/−	−/−	−/−	0	−/−	−/−	−/−	−/−	0	−/−	−/−	−/−	−/−	0
Direct PCR on water	−/−	−/−	−/NC	NC	0	−/−	−/−	−/−	−/−	0	−/−	−/−	−/−	−/−	0	−/−	−/−	−/−	−/−	0
PCR in isolated amoebae	−/−	+/+	+/NC	NC	60	−/−	−/−	+/+	±	37.5	−/−	±	−/−	±	25	NA/−	NA/NA	−/−	−/NA	0

*W, winter; Sp, spring; S, summer; A, autumn; NC, not collected; NA, not applicable (no isolated Acanthamoeba). Agar Culture and Direct PCR on water → free L. feeleii. PCR in isolated amoebae → Endosymbiont L. feeleii.*

**FIGURE 4:**
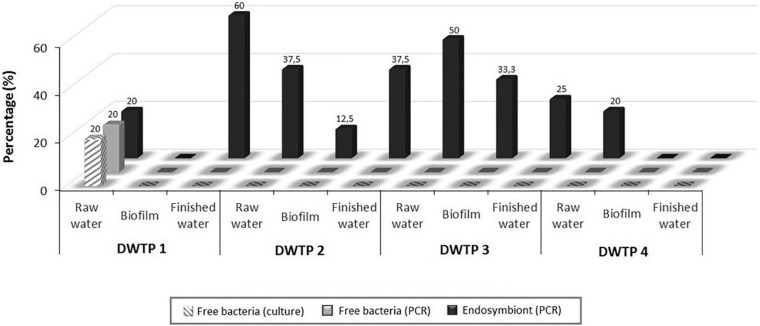
Presence of *L. feeleii* in each sampling point from 4 drinking water treatment plants (DWTPs) of the central region of Spain using different detection methodologies.

According to each DWTPs sampled, free *L. feeleii* was only identified at the entrance of the water treatment line of DWTP1 (raw water). On the other hand, the presence of FLA in DWTPs seemed to increase the presence of *L. feeleii* in raw water (20–50%) and finished water (0–60%). Regarding the decanting system, the development of biofilms in DWTPs 2 and 3 seemed to facilitate interaction between *L. feeleii* and FLA (Figure 4).

**FIGURE 5:**
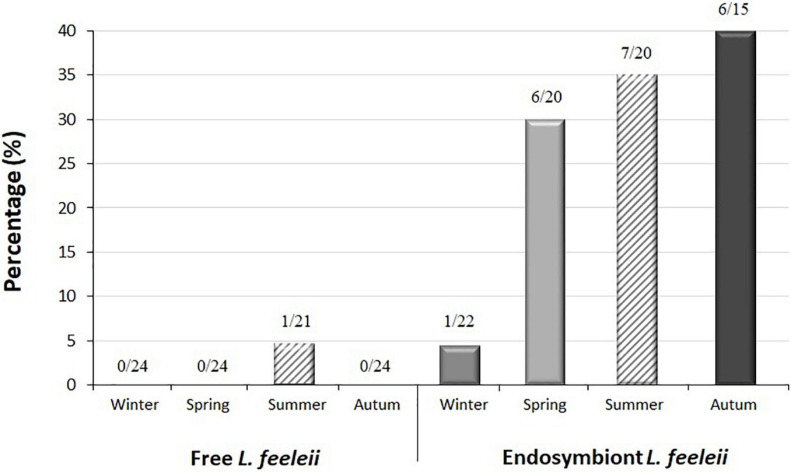
Presence of *L. feeleii* in finished water from drinking water treatment plants (DWTPs) according to season.

*L. feeleii* was found as a free-living form during summer. In contrast, the presence of *L. feeleii* increased when this bacterium was found inside *Acanthamoeba* spp., appearing in all seasons (Figure 5).

### Description of the First Case of Legionnaires’ Disease by *L. feeleii* in Spain

The first case of Legionnaires’ disease caused by *Legionella feeleii* in Spain was detected in the present study. The patient was a 75-year-old woman who attended a hospital in Madrid (Spain) in October 2013, presenting sudden onset of fever (38°C) with shivering of 48 h evolution, productive cough, dyspnea, and pleuritic chest pain. The patient had undergone ligation of esophageal varices, without complications, except for the appearance of pulmonary symptoms at 12 h after surgery. In addition, medical history revealed a diagnosis of diabetes mellitus type II in 1992 (insulin-dependent), hepatitis C virus genotype 1b infection in 1993 (untreated), and moderate mitral insufficiency.

**FIGURE 6:**
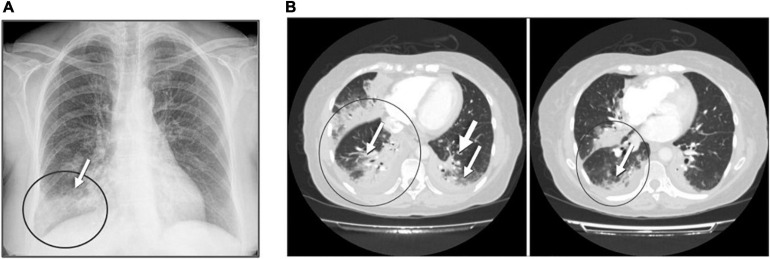
Admission chest radiography and computed tomography of the patient with szpneumonia caused by *L. feeleii*. **(A)** Chest radiography: radiological signs showed condensation in the lower right lobe (arrow) and a bilateral interstitial pattern. **(B)** Computed tomography showed extensive areas of consolidation with air bronchogram. Predominant affectation was observed in posterior basal segments and pseudo-nodular opacities of acinar aspect (arrows).

On physical examination, pulmonary auscultation revealed hypoventilation and crackling in the right lung. Chest radiography showed condensation at the base of the right lung (Figure 6A), and computed tomography (CT) of the chest showed an extensive area of consolidation with air bronchogram in bilateral posterior basal segments and the middle lobe, accompanied by pseudo-nodular opacities and other signs suggestive of inflammatory changes, as well as a small amount of bilateral pleural effusion (Figure 6B).

Laboratory analysis revealed a white blood cell count of 1.27 × 10^4^ cells/μL with 84% of neutrophil granulocytes, mild thrombocytopenia (8.6 × 10^4^ cells/μL), and a C-reactive protein level of 16.25 mg/L (reference value <5 mg/L). Arterial blood gas analysis showed decreased pO_2_ level (59 mmHg) and a 91% oxygen saturation (reference values 75–100 mmHg and 95–100%, respectively). Samples were collected for microbiological analysis, and the patient was treated empirically with ceftriaxone (2 g/day) and azithromycin (500 mg/day) through an intravenous line (IV), combined with a bronchodilator (Atrovent^®^ 500 μg/8 h). The following day, intravenous azithromycin was replaced orally.

Respiratory saprophytic microorganisms were isolated from sputum culture. *Legionella* and *Streptococcus pneumoniae* urinary antigen tests were negative. In order to establish the presence of *Legionella*, extraction of genomic DNA from urine and sputum samples was performed. Semi-nested PCR for amplification of *Legionella* spp. DNA (16S rRNA gene) was positive in the sputum sample. Also, a 447-bp DNA band was amplified from urine and sputum samples by conventional PCR targeting the *mip* gene of *L. feeleii.* The amplified product was sequenced, and alignment tests were carried out revealing a 99% similarity with *L. feeleii* (GenBank accession number AF023174). The sequence of the amplified product from urine and sputum samples was registered under accession number MW292583.

The patient evolved favorably, without complications, and 4 days post-admission, was discharged with oral antibiotic therapy at home (levofloxacin 500 mg/day) for 7 days. At follow-up, 1 month later, a chest x-ray showed resolution of the infection, with no infiltrates.

The Supplementary Table 2 summarizes this first case of Legionnaires’ disease caused *by L. feeleii* in Spain.

## Discussion

*Legionella feeleii* was described for the first time in 1981 during a Pontiac fever outbreak in an automobile assembly plant in Canada, in which a total of 317 workers were affected (Herwaldt et al., 1984). This *Legionella* species is a ubiquitous bacterium in the environment, and its role as an etiologic agent of Legionnaires’ disease has been previously demonstrated in other countries, mainly in immunocompromised patients (Supplementary Table 3). The mortality rate by *L. feeleii* infection can reach 28.6%, and it is the second most prevalent species of *Legionella* responsible for outbreaks of occupational Pontiac fever (39.4%) after *L. pneumophila* (58.5%) (Lee et al., 2009; Principe et al., 2016).

Nowadays, routine diagnosis of Legionnaires’ disease is carried out through urinary antigen detection (European Centre for Disease Prevention and Control, 2017). However, most commercial kits do not allow for detecting *L. pneumophila* non-serogroup 1 and other *Legionella* species (Fields et al., 2002). In a previous study, we found that this conventional method used in hospitals was only able to detect 4 out of 15 cases of pneumonia by *Legionella* (Vaccaro et al., 2016). Therefore, a low incidence of pneumonia by non-*L*. *pneumophila* could be attributed to the limitation of legionellosis diagnostic tools. PCR protocols could be helpful tools to improve legionellosis diagnosis, providing results within a short time and also detecting infections caused by other *Legionella* species and serogroups with high sensitivity and specificity (>90%) (Cloud et al., 2000; Murdoch, 2003; Svarrer and Uldum, 2012).

The PCR assay developed in this study for partial amplification of the *mip* gene has been demonstrated to be a sensitive and specific method for differential and simultaneous detection of some non-*L*. *pneumophila* species which can cause diseases in humans and can also be present in environmental sources, such as *L. feeleii* and *L. micdadei* (Yu et al., 2002; Kwon et al., 2011; Graham et al., 2012; Svarrer and Uldum, 2012). In addition to that, this assay could detect *L. tunisiensis*, a recently described environmental species (Campocasso et al., 2012; Pagnier et al., 2012). This PCR protocol enabled us to identify the first case of Legionnaires’ disease attributed to *L. feeleii* in Spain and improve the detection of this non-*L. pneumophila* in environmental samples.

In the present study, the environmental presence of *L. feeleii* was studied to perceive their impact on human infections. Samples from DWTPs of the central area of Spain were analyzed. Water purification treatments were shown to be effective for the elimination of free *L. feeleii* in the water. On the other hand, the presence of FLA in DWTPs, specifically *Acanthamoeba* spp., produced a significant rise of *L. feeleii* in warm weather and promoted the multiplication and survival of *Legionella* species at low temperatures (autumn and winter). The results may suggest that FLA are an important environmental reservoir for the amplification, persistence, and spread of this *Legionella* species from DTWPs to drinking water supply facilities. Taking into account that a high frequency of *Acanthamoeba* spp. has been described in other DWTPs (77–87%) and water sources (40–90%) from Spain, elimination strategies and control measures should be improved to remove FLA from DWTPs (Lorenzo-Morales et al., 2005; Magnet et al., 2012; Garcia et al., 2013).

The presence of *L. feeleii* in DTWPs may also depend on the origin of raw water and water purification treatments. For instance, microfiltration and ultrafiltration with membranes have been demonstrated to be less effective in reducing the presence of *Legionella*, while biofilm formation in water tanks could promote the proliferation of bacterial microorganisms (Lau and Ashbolt, 2009; Kwon et al., 2011). Our results also suggest that the interaction between *L. feeleii*-FLA increased in the sludge recirculation decanter system. With this exception, the water treatment line of DWTP4 was the most effective one for eliminating *L. feeleii* from biofilm and water samples. On the other hand, a higher biocide and amoebic effect are expected to occur during the distribution time due to the treatment with monochloramine (Flannery et al., 2006; Dupuy et al., 2011).

Moreover, the PCR protocol developed in this study was useful not only for environmental studies but also for detecting the first case of Legionnaires’ disease by *L. feeleii* in Spain that we have reported here. Molecular techniques allowed the detection of *L. feeleii* in urine and sputum samples from an elderly patient with pneumonia. The patient’s clinical condition included risk factors associated with *Legionella* spp. infection such as age (75-years old), diabetes, heart disease, and recent surgery. Additionally, other possible causes of pneumonia and respiratory pathogens were dismissed. In summary, we suggest that this pneumonia episode was a probable case of Legionnaires’ disease associated with health care facilities, according to the criteria established by the European Legionnaires’ Disease Surveillance Network and the Spanish Society of Pneumology and Thoracic Surgery (SEPAR) (Bartram et al., 2007; Blanquer et al., 2011).

In Spain, this represents the first reported case of pneumonia by *L. feeleii* and the first nosocomial Legionnaires’ disease produced by non-*L*. *pneumophila* species. Table 3 shows all reported cases of pneumonia caused by non-*Legionella pneumophila* in Spain to the present. Although Legionellosis cases are kept under surveillance by the autonomous communities of Spain and are weekly reported by the Red Nacional de Vigilancia Epidemiológica (RENAVE), the *Legionella* species associated with them is not described. The current description highlights the importance of a deeper study of Legionellosis disease cases as they might be underdiagnosed.

**TABLE 3:** Reported cases of pneumonia caused by non-*L. pneumophila* in Spain to the present.

**Year**	**Species**	**Source of infection**	**Age**	**Predisposing factors**	**Observations**
2000*	*L. longbeachae*	CAP	18	_	Immunocompetent patient. Severe pneumonia† (Ruiz et al., 2000)
2010*	*L. longbeachae*	CAP	61	_	Immunocompetent patient. Severe pneumonia (García-Somoza et al., 2010)
2011	*L. micdadei*	CAP	54	-Corticosteroids -Chronic kidney disease	Transplanted patient. Severe pneumonia (Soler-Majoral et al., 2016)
2014	*L. anisa*	CAP	36	_	Immunocompetent patient (Vaccaro et al., 2016)
2014	*L. feeleii*	Health care facilities	75	-Mitral insufficiency -Diabetes mellitus type II (insulin-dependent)	The patient underwent surgery Current case

*Date of publication. For these cases, the date of infection is unknown.

*CAP, community-acquired pneumonia; Ȃ, Fatal outcome.*

Furthermore, worldwide, it is the second case infection caused by *L. feeleii* associated with a surgical procedure (Lee et al., 2009). Nosocomial pneumonia has also been caused by other species of non-*L*. *pneumophila* from Intensive Care Units (ICU), endotracheal intubation, mechanical ventilation, and nasogastric tubes (Bornstein et al., 1989; Grove et al., 2002; Bartram et al., 2007).

Clinically, it is not possible to distinguish Legionnaires’ disease from other forms of pneumonia. Therefore, a proper diagnostic method that enables early identification of *Legionella* species is critical in the management of patients, as a delay in the provision of an effective treatment against *Legionella* could increase morbidity and mortality, especially in immunosuppressed patients (Ruiz et al., 2000; Pedro-Botet and Yu, 2006; Soler-Majoral et al., 2016; Chambers et al., 2021). Nowadays, Legionnaires’ disease diagnosis almost exclusively depends on urinary antigen tests, representing 82 and 97% of Legionnaires’ disease confirmation in Europe and the United States, respectively. In Spain, 97% of confirmed cases of legionellosis were diagnosed by this method in 2015 (Bartram et al., 2007; European Centre for Disease Prevention and Control, 2017). The popularity of urinary antigen tests can be attributed to their speed, relatively low cost, uncomplicated procedure, ease of sample collection, and commercial availability. However, most commercial kits can only detect *L. pneumophila* serogroup 1 (Muder and Yu, 2002; Blyth et al., 2009). For this reason, the introduction of PCR assay for the detection of different *Legionella* species has increased from 2.5% in 2010 to 10.5% in 2015 (European Centre for Disease Prevention and Control, 2017).

In 2015, the proportion of PCR ascertained cases was above 25% in some countries (Czech Republic, Sweden, and the United Kingdom) and 75% in Denmark (European Centre for Disease Prevention and Control, 2017). This molecular method is one of the most sensitive diagnostic tools for Legionnaires’ disease, able to provide results in a short time, detect different *Legionella* species simultaneously, and allow an increase in case of detection with greater sensitivity and specificity than reference techniques (culture and urinary antigen test) (Cloud et al., 2000; Murdoch, 2003; Lee et al., 2009; Murdoch et al., 2013; Avni et al., 2016). Higher sensitivity and specificity in detection were achieved in this study using a PCR protocol to amplify the *mip* gene of *L. feeleii* from clinical and environmental samples. Therefore, molecular techniques can be helpful tools for the diagnosis of legionellosis and the evaluation of their presence in the environment.

The detection of non-*L*. *pneumophila* is a factor that must be considered when establishing measures for control and prevention of legionellosis and when investigating the sources of infection giving rise to outbreaks since its presence in natural reservoirs and drinking water distribution systems may represent a risk factor.

## Data Availability Statement

The original contributions presented in the study are included in the article/Supplementary Material, further inquiries can be directed to the corresponding author/s.

## Ethics Statement

The studies involving human participants were reviewed and approved by the Research Ethics Committee of San Pablo CEU University. The patients/participants provided their written informed consent to participate in this study.

## Author Contributions

LV wrote the first draft of the manuscript. LV, TG, FI, AM, SL, DO, SS, AA, CG, CP, BB, SF, and CH-M contributed to collection of samples. LV, TG, FI, AM, BB, and CH-M performed the processing and analysis of the samples. ML contributed to the interpretation of clinical case and approved its publication. FI, CA, SF, and CH-M contributed to the conception and design of the work. CA and CH-M contributed to the funding acquisition. All authors contributed to manuscript revision, read, and approved the submitted version.

## Conflict of Interest

The authors declare that the research was conducted in the absence of any commercial or financial relationships that could be construed as a potential conflict of interest.

## Publisher’s Note

All claims expressed in this article are solely those of the authors and do not necessarily represent those of their affiliated organizations, or those of the publisher, the editors and the reviewers. Any product that may be evaluated in this article, or claim that may be made by its manufacturer, is not guaranteed or endorsed by the publisher.

## Abbreviations

DWTPs: drinking water treatment plants
FLA: free-living amoebae.
