# Supplementary material for: Legionella feeleii: Ubiquitous Pathogen in the Environment and Causative Agent of Pneumonia
Source: Front Microbiol. 2021 Aug 3;12:707187. doi: 10.3389/fmicb.2021.707187 (PMC8369763; doi:10.3389/fmicb.2021.707187)
Supplement: Supplementary file 1 [file Data_Sheet_1.pdf]

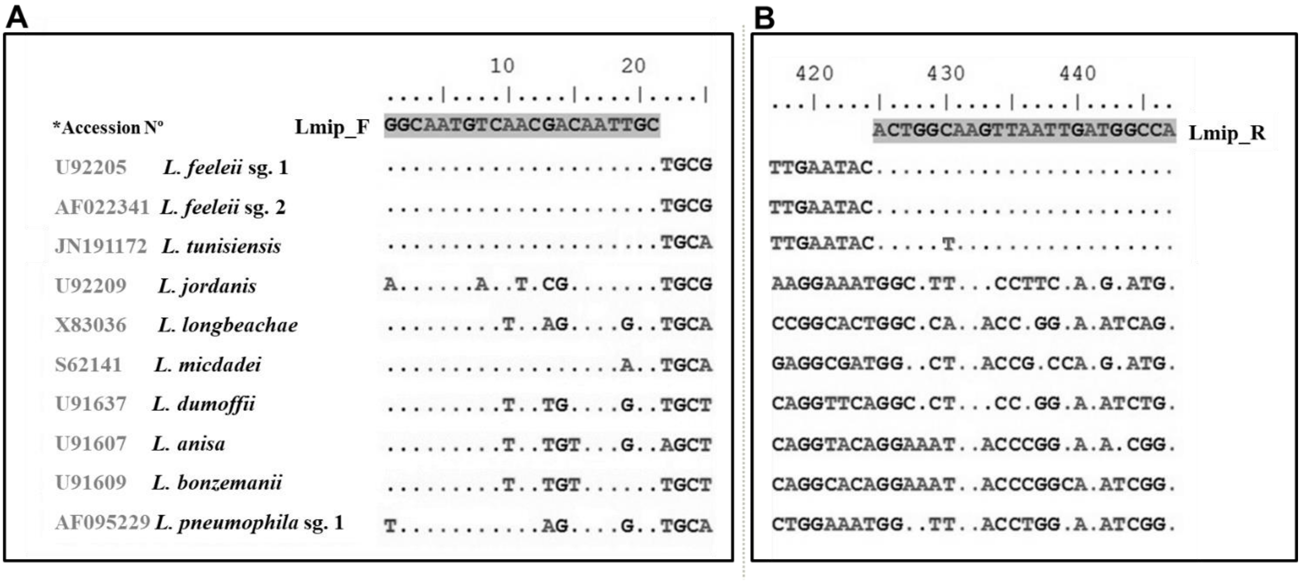

Supplementary Figure 1 Alignment of Lmip primers and DNA sequences of *mip* gene of *Legionella* species (ClustalW, Bioedit 7.2). These sequences were taken from the GenBank database. **(A)** Lmip_F primer. **(B)** Lmip_R primer. sg., serogroup. *Genbank accession number.
