# Supplementary material for: Legionella feeleii: Ubiquitous Pathogen in the Environment and Causative Agent of Pneumonia
Source: Front Microbiol. 2021 Aug 3;12:707187. doi: 10.3389/fmicb.2021.707187 (PMC8369763; doi:10.3389/fmicb.2021.707187)
Supplement: Supplementary file 2 [file Data_Sheet_2.pdf]

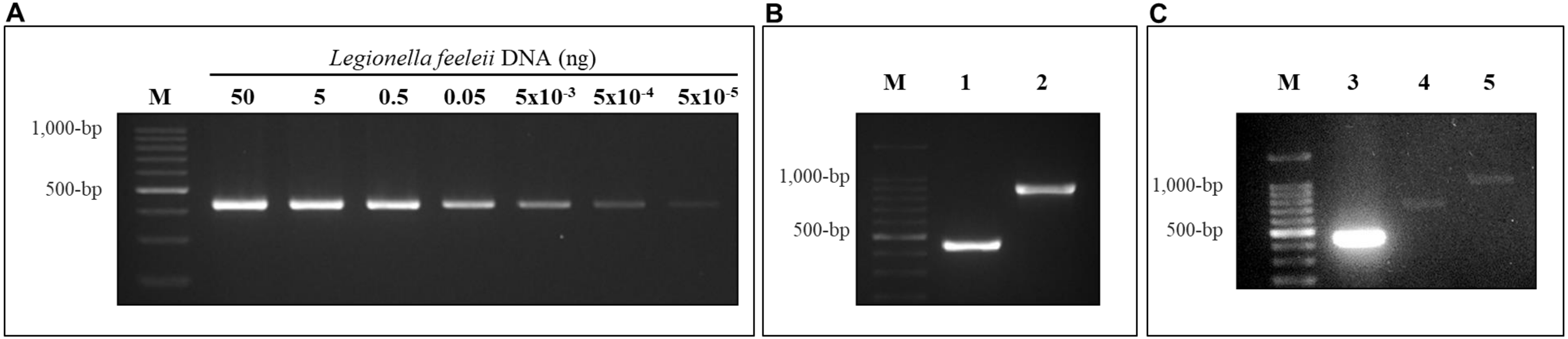

Supplementary Figure 2 Partial amplification of the *mip* gene of non-*Legionella pneumophila* species using a pair of Lmip primers. Electrophoresis in 2% agarose gels with ethidium bromide staining (0.5 µg/mL). **(A)** Limit of detection of DNA *L. feeleii* (ng). **(B)** Detection of DNA *L. feeleii* (line 1) and *L. micdadei* (line 2). **(C)** Detection of DNA *L. feeleii* (line 3), *L. longbeachae* (line 4) and *L. dumoffii* (line 5). M, DNA molecular weight marker (100-bp ladder). *L. feeleii* was isolated from environmental sample by Magnet et al., 2015. *L. micdadei*: ATCC33218. *L longbeachae:* ATCC33462. *L. dumoffii*: ATCC33279.
