# Supplementary material for: Legionella feeleii: Ubiquitous Pathogen in the Environment and Causative Agent of Pneumonia
Source: Front Microbiol. 2021 Aug 3;12:707187. doi: 10.3389/fmicb.2021.707187 (PMC8369763; doi:10.3389/fmicb.2021.707187)
Supplement: Supplementary file 3 [file Data_Sheet_3.pdf]

```
                          10        20        30        40        50        60        70        80        90       100
                  ....|....|....|....|....|....|....|....|....|....|....|....|....|....|....|....|....|....|....|....|
Lmip_F            GGCAATGTCAACGACAATTGC
L. feeleii sg. 1  GGCAATGTCAACGACAATTGCTGCGGCTGATGCGACAGCTACAACATCATTAAATAGTGACATGGATAAGTTGTCTTATAGCATTGGCGCTGATTTAGGT
L. feeleii sg. 2  GGCAATGTCAACGACAATTGCTGCGGCTGATGCGACAGCTACAACATCATTAAATAGTGACATGGATAAGTTGTCTTATAGCATTGGCGCTGATTTAGGT
L. tunisiensis    GGCAATGTCAACGACAATTGCTGCAGCTGATGCGGCAACTACAACATCATTAAATAGTGACATGGATAAGTTGTCTTATAGCATTGGCGCTGATTTAGGT
Lmip_R            ....................................................................................................

                         110       120       130       140       150       160       170       180       190       200
                  ....|....|....|....|....|....|....|....|....|....|....|....|....|....|....|....|....|....|....|....|
Lmip_F
L. feeleii sg. 1  AAGAATTTTAAAAAGCAAGGCATCGAAATCAGTCCGGCTGCTATGGCTAAGGGATTACAAGACGGCATGAGTGGTGGTCAACTGTTGCTGACTGAACAAC
L. feeleii sg. 2  AAGAATTTTAAAAAGCAAGGCATCGAAATCAGTCCGGCTGCTATGGCTAAGGGATTACAAGACGGCATGAGTGGTGGTCAACTGTTGCTGACTGAACAAC
L. tunisiensis    AAGAATTTTAAGAAGCAAGGTATCGACATCAGTCCTGCTGCTATGGCTAAGGGATTACAAGACGGCATGAGTGGTGGTCAACTGTTGCTGACTGAACAAC
Lmip_R            ....................................................................................................

                         210       220       230       240       250       260       270       280       290       300
                  ....|....|....|....|....|....|....|....|....|....|....|....|....|....|....|....|....|....|....|....|
Lmip_F
L. feeleii sg. 1  AAATGAAAGACGTTTTAAATAAATTTCAAAAAGACCTAATGGCAAAACGAAATGCGGAATTCACTAAAAAAGCTGAAGAAAATAAAGCGAAAGGTGAAGC
L. feeleii sg. 2  AAATGAAAGACGTTTTAAATAAATTTCAAAAAGACCTAATGGCAAAACGAAATGCTGAATTCACTAAAAAAGCTGAAGAAAATAAAGCGAAAGGTGAAGC
L. tunisiensis    AAATGAAAGATGTTTTAAATAAGTTTCAAAAAGACCTAATGGCAAAGCGAAATGCTGAATTCACCAAAAAAGCTGAAGAGAACAAAGCTAAAGGTGAAGC
Lmip_R            ....................................................................................................

                         310       320       330       340       350       360       370       380       390       400
                  ....|....|....|....|....|....|....|....|....|....|....|....|....|....|....|....|....|....|....|....|
Lmip_F
L. feeleii sg. 1  GTTCATGAGCCAGAATAAGGCCAAAGAAGGCGTAGTTAGTTTGCCAAGCGGGTTACAATATAAAATAATCCAGGCTGGTACTGGTGCAAAACCAGCAAAA
L. feeleii sg. 2  GTTCATGAGCCAGAATAAGGCCAAAGAAGGCGTAGTTAGTTTGCCAAGCGGGTTACAATATAAAATAATCCAGGCTGGTACTGGTGCAAAACCAGCAAAA
L. tunisiensis    ATTCATGAGCCAAAACAAGGCTAAAGAAGGTGTAGTCAGTTTGCCAAGTGGATTGCAATACAAAGTAATCCAAACGGGTAGTGGTGCAAAGCCCGCGAAA
Lmip_R            ....................................................................................................

                         410       420       430       440
                  ....|....|....|....|....|....|....|....|....|..
Lmip_F
L. feeleii sg. 1  GATGACACTGTCACTGTTGAATACACTGGCAAGTTAATTGATGGCCA
L. feeleii sg. 2  GATGACACTGTTACTGTTGAATACACTGGCAAGTTAATTGATGGCCA
L. tunisiensis    GATGACACTGTCACTGTTGAATACACTGGTAAGTTAATTGATGGCCA
Lmip_R            ........................ACTGGCAAGTTAATTGATGGCCA
```

Supplementary Figure 3 Alignment of DNA sequences of *mip* gene of *L. feeleii* and *L. tunisiensis (*Bioedit 7.2). sg., serogroup. GenBank accession number: *L. feeleii* sg. 1 (U92205), *L. feeleii* sg. 2 (AF022341), *L. tunisiensis* (JN191172).
