# Supplementary material for: Legionella feeleii: Ubiquitous Pathogen in the Environment and Causative Agent of Pneumonia
Source: Front Microbiol. 2021 Aug 3;12:707187. doi: 10.3389/fmicb.2021.707187 (PMC8369763; doi:10.3389/fmicb.2021.707187)
Supplement: Supplementary file 4 [file Data_Sheet_4.pdf]

Supplementary Table 1 Amplification of a 447-bp DNA fragment from other respiratory pathogens.

Pathogen	Strain from	PCR product 447-bp
Acromobacter xylosoxidans	environmental specimen	Negative
Aspergillus fumigatus	clinical specimen	Negative
Candida albicans	clinical specimen	Negative
Escherichia coli	clinical specimen	Negative
Haemophilus influenzae	clinical specimen	Negative
Klebsiella pneumoniae	clinical specimen	Negative
Mycobacterium spp.	clinical specimen	Negative
Mycoplasma spp.	control kit	Negative
Pseudomonas aeruginosa	environmental specimen	Negative
Staphylococcus aureus	clinical specimen	Negative
Stenotrophomonas maltophilia	environmental specimen	Negative
Streptococcus pneumoniae	clinical specimen	*

* *S. pneumoniae* presented a double-banded pattern with DNA amplification at 450 and 800-bp.
