# Supplementary material for: Legionella feeleii: Ubiquitous Pathogen in the Environment and Causative Agent of Pneumonia
Source: Front Microbiol. 2021 Aug 3;12:707187. doi: 10.3389/fmicb.2021.707187 (PMC8369763; doi:10.3389/fmicb.2021.707187)
Supplement: Supplementary file 5 [file Data_Sheet_5.pdf]

Supplementary Table 2 Detailed description of the first case of Legionnaires' disease by *L. feeleii* in Spain diagnosed in this study.

Medical history	Signs and symptoms	Image and laboratory analysis	Treatment and progress
-75-year-old woman.<br>-Diabetes mellitus type II (insulin-dependent).<br>-Hepatitis C virus (untreated).<br>-Moderate mitral insufficiency.	Symptoms appeared 12 hours after surgery.<br>-Fever (38°C).<br>-Shivering.<br>-Productive cough.<br>-Dyspnea.<br>-Pleuritic chest pain.	**Image analysis:**<br>-<u>Chest radiography:</u> condensation at the base of the right lung.<br>-<u>Computed tomography:</u> an extensive area of consolidation with air bronchogram in bilateral posterior basal segments and the middle lobe, pseudo-nodular opacities and other signs suggestive of inflammatory changes, a small amount of bilateral pleural effusion.	**Treatment:**<br>-Ceftriaxone (2 g/day) and azithromycin (500 mg/day) by IV. The following day, intravenous azithromycin was replaced orally.<br>-Atrovent® (bronchodilator) 500 µg/8 hours.<br>-Oral therapy at home: levofloxacin (500 mg/day) for 7 days.
	**Physical exam:**<br>-Pulmonary auscultation revealed hypoventilation and crackling in the right lung.	**Laboratory results:**<br>-Neutrophilia.<br>-High CRP level.<br>-Decreased pO_2 level and oxygen saturation.	**Evolution:**<br>-Favorable, without complications.<br>-Discharge: four days post-admission.<br>-1 month later: a chest x-ray showed resolution of the infection, with no infiltrates.
		**Microbiological analysis:**<br>-<u>Sputum culture:</u> Respiratory saprophytic microorganisms.<br>-<u>Urinary antigen tests:</u><br>**Legionella:* negative.<br>**Streptococcus pneumoniae:* negative.	
		**Molecular diagnostics:**<br>-<u>Samples</u>: urine and sputum.<br>-<u>PCR for amplification of *Legionella:*</u><br>*Semi-nested PCR (16S rRNA gene): positive.<br>*Conventional PCR (*mip* gene): positive.<br>-<u>Sequencing analysis:</u><br>*L. feeleii* (GenBank accession number MW292583).	

CRP, C-reactive protein; PCR, polymerase chain reaction; IV, intravenous line.
