# Supplementary material for: Legionella feeleii: Ubiquitous Pathogen in the Environment and Causative Agent of Pneumonia
Source: Front Microbiol. 2021 Aug 3;12:707187. doi: 10.3389/fmicb.2021.707187 (PMC8369763; doi:10.3389/fmicb.2021.707187)
Supplement: Supplementary file 6 [file Data_Sheet_6.pdf]

Supplementary Table 3 Cases of legionellosis attributed to *L. feeleii* worldwide from 1981 to the present.

Year	Country	Number of cases	Serogroup (sg.)	Form of illness	Source of infection	Observations
1981	Canada	317	sg. 1	PF	CAP	The first cases reported worldwide (Herwaldt et al., 1984)
1985	USA	2	sg. 2	LD	Unknown	Identification of *L. feeleii* serogroup 2 (Thacker et al., 1985)
1986*	USA	2	sg. 1	LD	CAP	Immunosuppressed patients ł (Palutke et al., 1986)
1987*	USA	1	Unknown	LD	CAP	ł (Misra et al., 1987)
1992	France	1	sg. 1	LD	Nosocomial	First case in Europe ł (Lo Presti et al., 1998)
1994	New Zealand	1	sg. 2	LD	CAP	(Schousboe et al., 1995)
1996	France	1	sg. 1	LD	CAP	Immunosuppressed patient (Lo Presti et al., 1998)
1997*	Israel	1	Unknown	LD and pericarditis	CAP	First case of extrapulmonary infection (Sviri et al., 1997)
2002*	Unknown	2	Unknown	LD	CAP	An international collaborative survey (Yu et al., 2002)
2008*	Unknown	1	Unknown	LD	Nosocomial	Immunosuppressed patient (Jacobson et al., 2008)
2009	France	1	Unknown	Cellulitis	Unknown	Cutaneous lesion (Loridant et al., 2011)
2009*	USA	1	Unknown	LD	Unknown	Transplanted patient (Lee et al., 2009)
2010*	USA	1	sg. 2	LD	Unknown	Immunosuppressed patient (Siegel et al., 2010)
2015*	USA	1	sg. 2	LD	Unknown	Immunosuppressed patient ł (Han et al., 2015)
2018	United Kingdom	1	Unknown	Cutaneous eruption	Unknown	Immunosuppressed patient (Verykiou et al., 2018)
2018*	Japon	1	Unknown	Unknown	Unknown	*Legionella* clinical isolates (2008-2016) (Amemura-Maekawa et al., 2018)

*Date of publication. For these cases, the date of infection is unknown. PF, Pontiac fever; LD, Legionnaires' disease; CAP, community-acquired pneumonia; ł Fatal outcome.

REFERENCES

Amemura-Maekawa, J., Kura, F., Chida, K., Ohya, H., Kanatani, JI., Isobe, J. et al. (2018). Legionella pneumophila and Other Legionella Species Isolated from Legionellosis Patients in Japan between 2008 and 2016. Applied and environmental microbiology. 84. https://doi.org/10.1128/AEM.00721-18

Han, XY., Ihegword, A., Evans, SE., Zhang, J., Li, L., Cao, H., et al. (2015). Microbiological and Clinical Studies of Legionellosis in 33 Patients with Cancer. J Clin Microbiol, in press. https://doi.org/10.1128/JCM.00380-15

Herwaldt, LA., Gorman, GW., McGrath, T., Toma, S., Brake, B., Hightower, AW., et al. (1984). A new Legionella species, Legionella feeleii species nova, causes Pontiac fever in an automobile plant. Ann Intern Med. 100, 333-338. https://doi.org/10.7326/0003-4819-100-3-333

Jacobson, KL., Miceli, MH., Tarrand, JJ., and Kontoyiannis, DP. (2008). Legionella pneumonia in cancer patients. Medicine (Baltimore). 87, 152-159. https://doi.org/10.1097/MD.0b013e3181779b53

Lee, J., Caplivski, D., Wu, M., and Huprikar, S. (2009). Pneumonia due to Legionella feeleii: case report and review of the literature. Transplant infectious disease: an official journal of the Transplantation Society. 11, 337-340. https://doi.org/10.1111/j.1399-3062.2009.00390.x

Lo Presti, F., Riffard, S., Neyret, C., Célard, M., Vandenesch, F., and Etienne, J. (1998). First isolation in Europe of Legionella feeleii from two cases of pneumonia. European journal of clinical microbiology & infectious diseases : official publication of the European Society of Clinical Microbiology. 17, 64-66. https://doi.org/10.1007/BF01584371

Loridant, S., Lagier, J., and La Scola, B. (2011). Identification of Legionella feeleii cellulitis. Emerg Infect Dis. 17, 145-146. https://doi.org/10.3201/eid1701.101346

Misra, D., Harris, L., and Shasteen, W. (1987). Legionella feeleii pneumonia. South Med J. 80, 1063-1064. https://doi.org/10.1097/00007611-198708000-00035

Palutke, WA., Crane, LR., Wentworth, BB., Geiger, JG., Cardozo, L., Singhakowinta, A., et al. (1986). Legionella feeleii-associated pneumonia in humans. American journal of clinical pathology. 86, 348-351. https://doi.org/10.1093/ajcp/86.3.348

Schousboe, M., Gibbons, S., and Chereshsky, A. (1995). Community-acquired pneumonia due to Legionella feeleii serogroup 2. N Z Med J. 108, 279. https://doi.org/10.1128/JCM.00176-10.

Siegel, MO., Fedorko, DP., Drake, SK., Calhoun, LB., and Holland, SM. (2010). Legionella feeleii serotype 2 pneumonia in a man with chronic lymphocytic leukemia: a challenging diagnosis. J Clin Microbiol. 48, 2294-2297. https://doi.org/10.1128/JCM.00176-10

Sviri, S., Raveh, D., Boldur, I., Safadi, R., Libson, E., and Ben-Yehuda, A. (1997). Legionella feeleii pneumonia and pericarditis. The Journal of infection. 34, 277-279. https://doi.org/10.1016/s0163-4453(97)94535-7

Thacker, WL., Wilkinson, HW., Plikaytis, BB., Steigerwalt, AG., Mayberry, WR., Moss, CW., et al. (1985). Second serogroup of Legionella feeleii strains isolated from humans. J Clin Microbiol. 22, 1-4. https://doi.org/10.1128/JCM.22.1.1-4.1985

Verykiou, S., Goodhead, C., Parry, G., and Meggitt, S. (2018). Legionella feeleii: an unusual organism associated with cutaneous infection in an immunocompromised patient. Clin Exp Dermatol. 43, 300-302. https://doi.org/10.1111/ced.13346

Yu, VL., Plouffe, JF., Pastoris, MC., Stout, JE., Schousboe, M., Widmer, A., et al. (2002). Distribution of Legionella species and serogroups isolated by culture in patients with sporadic community-acquired legionellosis: an international collaborative survey. J Infect Dis. 186, 127-128. https://doi.org/10.1086/341087
